# A patient-specific planning target volume used in ‘plan of the day’ adaptation for interfractional motion mitigation

**DOI:** 10.1093/jrr/rrt070

**Published:** 2013-07

**Authors:** Wenjing Chen, Alexander Gemmel, Eike Rietzel

**Affiliations:** 1 UniversitätsKlinikum Heidelberg, Radiologische Klinik, Im Neuenheimer Feld 400, 69120, Heidelberg, Germany; 2 Siemens Healthcare Sector, Imaging & Therapy Division, Particle Therapy, Hofmannstraße 26, 91052, Erlangen, Germany

**Keywords:** particle therapy, treatment planning, organ motion, prostate, planning target volume

## Abstract

We propose a patient-specific planning target volume (PTV) to deal with interfractional variations, and test its feasibility in a retrospective treatment-planning study. Instead of using one planning image only, multiple scans are taken on different days. The target and organs at risk (OARs) are delineated on each images. The proposed PTV is generated from a union of those target contours on the planning images, excluding voxels of the OARs, and is denoted the PTV ‘GP–OAR’ (global prostate–organs at risk). The study is performed using ‘plan of the day’ adaptive workflow, which selects a daily plan from a library of plans based on a similarity comparison between the daily scan and planning images. The daily plans optimized for GP–OAR volumes are compared with those optimized for PTVs generated from a single prostate contour (PTV SP). Four CT serials of prostate cancer patient datasets are included in the test, and in total 28 fractions are simulated. The results show that the daily chosen GP–OAR plans provide excellent target coverage, with V95 values of the prostate mostly > 95%. In addition, dose delivered to the OARs as calculated from applying daily chosen GP–OAR plans is slightly increased but comparable to that calculated from applying daily SP plans. In general, the PTV GP–OARs are able to cover possible target variations while keeping dose delivered to the OARs at a similar level to that of the PTV SPs.

## INTRODUCTION

Particle therapy has been proven to be an effective treatment option for several tumor types [1–4], and the prostate is one of the major treatment sites [5–7]. Nevertheless, uncertainties such as setup errors and organ motions can occur during a treatment course, influencing dose distribution and impairing treatment effect and healthy tissue compliance [8–10]. To cover those potential variations, additional margins are applied to the clinical target volume (CTV). On the other hand, this inevitably leads to an increase in the dose delivered to the surrounding normal tissues [11, 12]. To achieve margin reduction while retaining dose coverage for targets, various image guidance and adaptive strategies have been investigated [13–15]. A common clinical practice is patient repositioning based on the registration between daily and planning images. The drawback is that this might not be able to cover large organ deformation [16]. To compensate for interfractional variations for individual patients, there have been studies made on the patient-specific planning target volume (PTV) generated from multiple images [17, 18]. In this study, we investigate a patient-specific PTV, which takes not only the target but also OARs into consideration. A union of target contours on different images is formed to cover possible positions and volume changes of the target, and the voxels that belong to the OARs are excluded from the union to restrict the dose delivered to the OARs. The remaining part is the proposed PTV, and is denoted the ‘GP–OAR’ volume. The feasibility study is performed using the ‘plan of the day’ (PoD) workflow. Several groups have conducted research with this adaptive method in the pelvic region [19–22]: a multiple-plan library is generated before the treatment course starts, and during each fraction a daily plan is manually chosen from the library according to the patient's daily anatomy. In our study, we perform similarity comparisons between the daily scan and the planning images in order to automatically select the PoD. The effectiveness of motion mitigation of the PTV GP–OAR is tested using prostate cancer patient datasets retrospectively, and compared with that of the PTV generated from a single prostate contour (PTV SP). The two types of daily chosen plans are compared with one another in terms of target coverage and OAR sparing.

## MATERIALS AND METHODS

### Patient datasets

This retrospective planning study included four prostate cancer patients, each with a weekly CT serial of 6–8 datasets. The prostate CTV, bladder and rectum were contoured on each CT scan. Mean sizes and volume variations of the organs are listed in Table 1.

**Table 1.** Number of CT datasets of each patient, mean and standard deviation of prostate/bladder/rectum gas volumes, and volume variation calculated from dividing standard deviation by the mean volume

Patient	P1	P2	P3	P4
Number of CT datasets	7	7	6	8
Mean target volume (cm^3^)	70/148/10	50/107/2	26/110/3	41/410/2
Standard deviation (cm^3^)	4/72/10	11/46/3	2/27/2	9/132/3
Volume variation (%)	6/49/100	23/43/150	6/25/67	22/32/150

### Treatment planning volumes

**Fig. 1.**
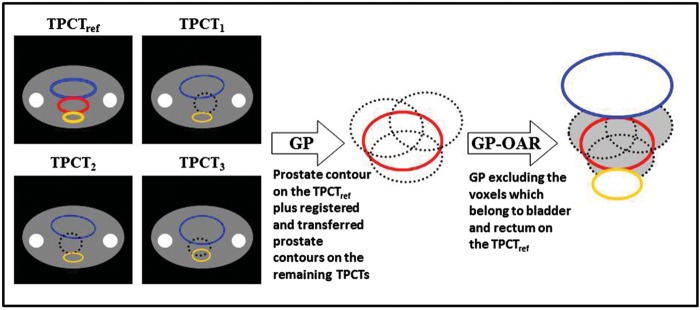
Generation of a GP–OAR volume. Bladder = blue contour, rectum = yellow contour, prostate on TPCTref = red contour, prostate on the other TPCTs = black dashed contour, global prostate (GP) volume = combination of the prostate contour on the TPCTref and the rigidly transformed prostate contours on the remaining TPCTs, GP–OAR volume = global prostate excluding the voxels of the OARs on the TPCTref (shown in gray in the right subplot).

The proposed PTV is generated on a reference planning image from a union of prostate contours, excluding voxels belonging to the OARs. As shown in Fig. 1, one treatment planning CT dataset is taken as the reference (TPCTref) from several available planning images (TPCTs). The remaining TPCTs are rigidly registered to the TPCTref, and the target contours are transformed onto the TPCTref according to the registration matrices. These contours and the target contour on the TPCTref are overlaid with one another, and the union forms a global prostate (GP) volume. In order to reduce the dose delivered to the OARs, voxels that belong to the bladder and the rectum on the TPCTref are excluded from the GP volume. The remaining part plus a 6-mm margin is denoted the PTV GP–OAR.

To demonstrate the effectiveness of target coverage of the proposed GP–OAR volume, an additional PTV SP is generated for comparison. It is formed in the usual manner, which is from one target volume plus additional margins. In order to compare OAR sparing between the two PTVs, the margins of the SP volumes are set as 6 mm, as are the GP–OAR volumes.

### Treatment planning

Carbon ion plans are optimized for each patient dataset by TRiP98 [23]. As a proof-of-principle study, only physical plans are optimized, and the relative biological effectiveness is not taken into consideration. The lateral distance between pencil-beam positions is 2 mm, the longitudinal distance is roughly 3 mm in water equivalent path length, and the beam focus size is chosen as 6 mm. Plans are generated on both PTV GP–OARs (GP–OAR plans) and PTV SPs (SP plans). A fractional absorbed dose of 1 Gy is delivered homogeneously to a PTV using two lateral opposing beams (+90° and –90°). To ensure sufficient dose coverage of a target, plans are accepted when the target volume percentage, which receives at least 95% of the prescribed dose (V95), is higher than 95%. In addition, the volume percentage of the OARs exposed to 50% or more of the prescribed dose (V50) is restricted below 50%.

### PoD adaptation

**Fig. 2.**
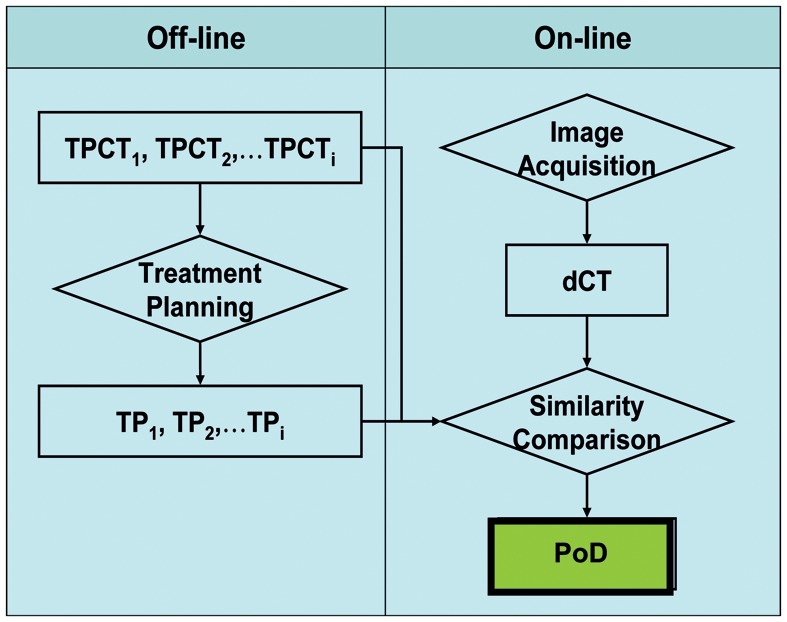
Treatment ‘plan of the day’ workflow.

The retrospective treatment-planning study follows the PoD adaptive workflow (see Fig. 2): before a treatment course starts, a plan library (TP1, TP2, … TPi) is generated from multiple images (TPCT1, TPCT2, … TPCTi) taken on different days. During each fraction, a daily position verification image (dCT) is taken. The dCT is rigidly registered to the TPCTs based on bony structure alignment. Mutual information (MI) calculated between the registered daily image and planning images facilitates the PoD selection. The planning image, which has the highest MI compared with the daily image, is identified and the associated plan is applied to the patient as the PoD.

### Treatment fraction simulation

To make the most use of the four CT serials for the feasibility study, each scan of a patient is picked as a daily image and a single fraction is simulated. The other scans in the CT serial are seen as pre-taken treatment-planning images. Two plans are optimized on each planning image with both PTVs, and a GP–OAR plan library and an SP plan library are generated for each simulated fraction. According to the adaptive workflow described above in *PoD adaptation*, a PoD is chosen from each of the plan libraries and dose calculation is performed on the daily scan.

### Evaluation method

To demonstrate the effectiveness of the proposed adaptive workflow, the chosen PoD is compared with other plans in the library. The V95 of the target, V50 and V70 of the OARs are derived from the dose volume histograms (DVHs). The plan that gives the highest V95 of the target is seen as the ‘best plan’ in the library for each simulated fraction. When the chosen PoD is the best plan, or the V95 difference of the target calculated between applying the best plan and the PoD is less than 5%, the plan selection is considered to be acceptable. In addition, the daily chosen GP–OAR plans (PoDs_GP–OAR_) are compared with the daily chosen SP plans (PoDs_SP_) in terms of target coverage and dose delivered to the OARs.

## RESULTS

### Target coverage

**Fig. 3.**
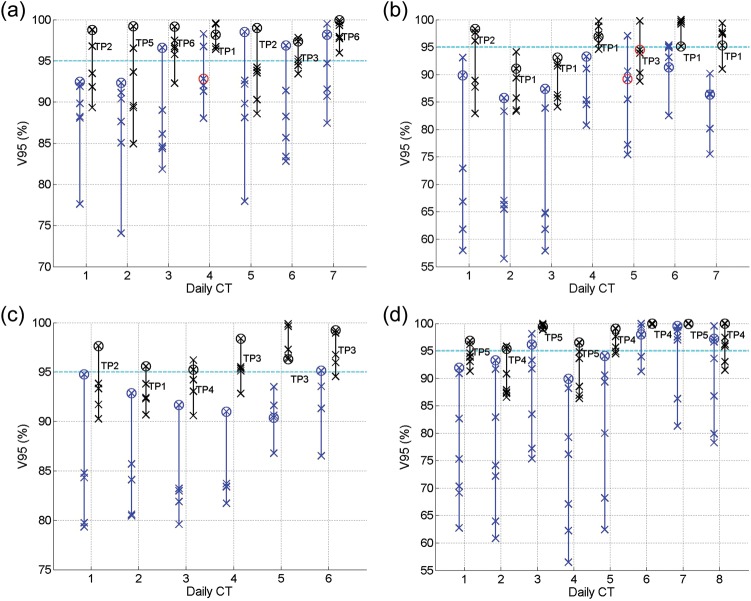
V95 values of the prostates calculated from applying all SP plans (blue crosses) and GP–OAR plans (black crosses) in the libraries of (**a**) patient 1; (**b**) patient 2; (**c**) patient 3; (**d**) patient 4. Daily chosen PoDs are marked with circles. When V95 differences for a target calculated between applying the best plans and the PoDs are >5%, the circles are shown in red.

Figure 3 illustrates the V95 values of the targets calculated from applying all the SP plans (blue crosses) and GP–OAR plans (black crosses) in the SP and GP–OAR libraries, respectively. Daily plans PoDs_SP_ and PoDs_GP–OAR_ are marked with circles in blue and black, respectively. When V95 calculated differences between applying a PoD and the best plan in a library are > 5%, the colour of the marker circle is changed into red. The V95 calculated differences for the targets between applying the best GP–OAR plans and the daily chosen PoDs_GP–OAR_ are < 5% in all but one case. In daily case 5 of patient 2, the V95 difference between the two plans is 5.3%. For each patient, the V95 range of a target when applying all GP–OAR plans in the library varies from daily case to daily case, with the maximum range of 14.3%, 15.4%, 7.4% and 10.2% for patients 1, 2, 3 and 4, respectively. In the worst case scenarios, the V95 values of the targets are 84.9%, 82.9%, 90.3% and 86.3% when applying a random GP–OAR plan for the four patients, respectively. On the other hand, if chosen by MI, calculated by comparing the daily scan with the planning images, in 25 out of a total of 28 daily cases the V95 of the target calculated from applying the chosen PoD_GP–OAR_ is > 95%. In the exceptional daily cases 2, 3 and 5 of patient 2, the V95 values of the target are 91.1%, 93.1% and 94.5%, respectively. Comparing PoDs_SP_ to the other SP plans, in all cases but two the V95 differences of the prostates calculated by comparing the best SP plans with the PoDs_SP_ are < 5%. In daily case 4 of patient 1 and daily case 5 of patient 2, the differences are 5.5% and 7.9%, respectively. The V95 range of the whole SP plan library varies, and the maximum ranges in all daily cases are 20.6%, 35.1%, 15.4% and 33.4% for patients 1, 2, 3 and 4, respectively. The V95 values for the targets calculated from applying a random SP plan can drop to 74.1%, 56.5%, 79.3% and 56.5% in the worst cases scenarios for the four patients, respectively. Chosen by similarity comparison, the daily PoDs_SP_ are always able to provide V95 values of the prostates > 90% for patients 1, 3 and 4, and > 85% for patient 2. There are nine out of 28 simulated daily cases in which the V95 values of the targets calculated from applying the PoDs_SP_ are able to achieve 95%. To compare target coverage of daily chosen PoDs_GP–OAR_s and PoDs_SP_, V95 differences of the prostates between applying the two types of plans are calculated and listed in Table 2. The mean improvements in V95 of the targets when applying the PoDs_GP–OAR_ are 3.4%, 5.9%, 4.4% and 3.4% for patients 1, 2, 3 and 4, respectively. In 13 out of 28 cases the improvements are > 5%, with the maximum differences of 6.9%, 9.0%, 7.4% and 6.6% for the four patients, respectively.

**Table 2.** V95 difference (%) of the target calculated between applying PoD_GP–OAR_ and PoD_SP_

Daily case	1	2	3	4	5	6	7	8	Mean
**Patient 1**	6.3	6.9	2.6	5.4	0.5	0.5	1.8		3.4
**Patient 2**	8.4	5.4	5.8	3.6	5.3	3.8	9.0		5.9
**Patient 3**	2.9	2.7	3.6	7.4	5.9	4.1			4.4
**Patient 4**	4.9	2.1	3.3	6.6	5.0	2.0	0.4	2.7	3.4

### Dose to the OARs

**Fig. 4.**
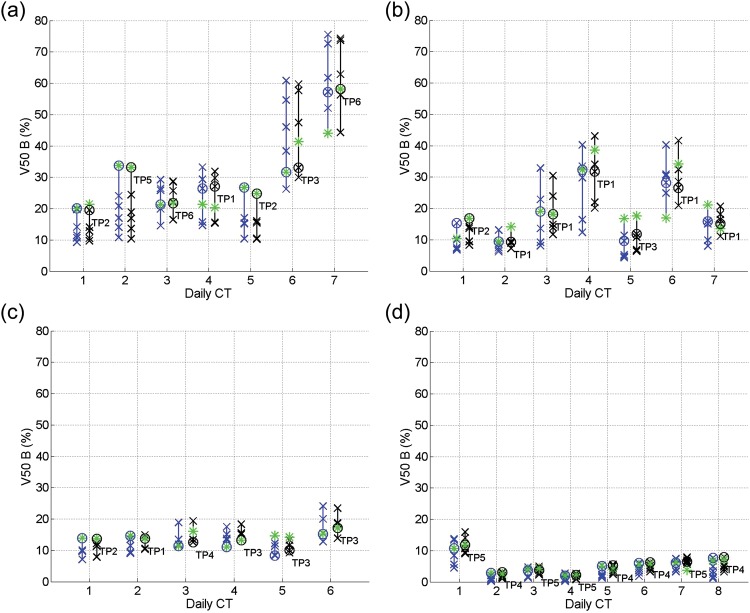
V50 values for the bladder calculated from applying all the SP plans (blue crosses) and GP–OAR plans (black crosses) in the libraries of (**a**) patient 1, (**b**) patient 2, (**c**) patient 3, and (**d**) patient 4. Daily chosen PoDs are marked with circles, and the best plans in the libraries are marked with asterisks.

**Fig. 5.**
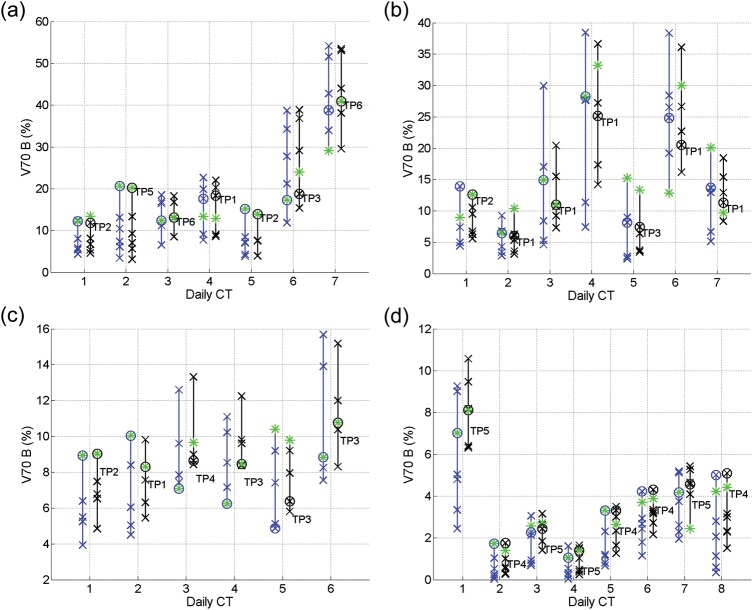
V70 values of the bladders calculated from applying all the SP plans (blue crosses) and GP–OAR plans (black crosses) in the libraries of (**a**) patient 1, (**b**) patient 2, (**c**) patient 3, and (**d**) patient 4. Daily chosen PoDs are marked with circles, and the best plans in the libraries are marked with asterisks.

**Fig. 6.**
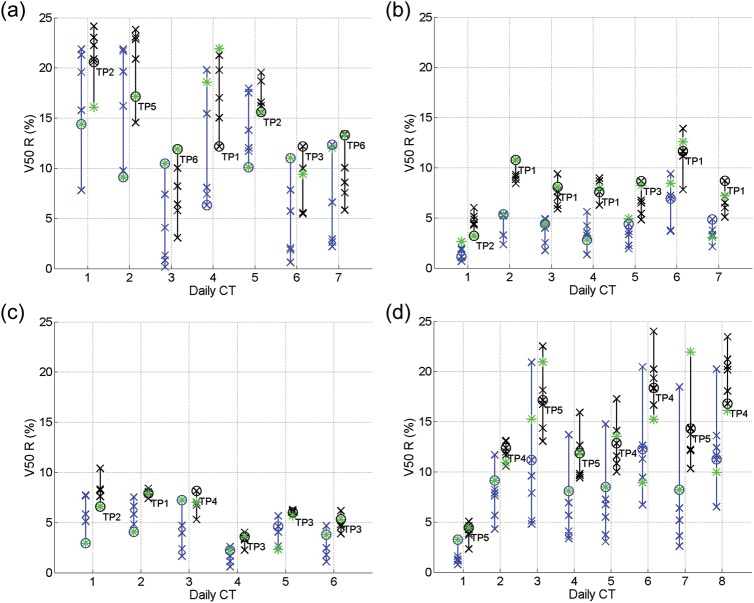
V50 values for the rectum calculated from applying all the SP plans (blue crosses) and GP–OAR plans (black crosses) in the libraries of (**a**) patient 1, (**b**) patient 2, (**c**) patient 3, and (**d**) patient 4. Daily chosen PoDs are marked with circles, and the best plans in the libraries are marked with asterisks.

**Fig. 7.**
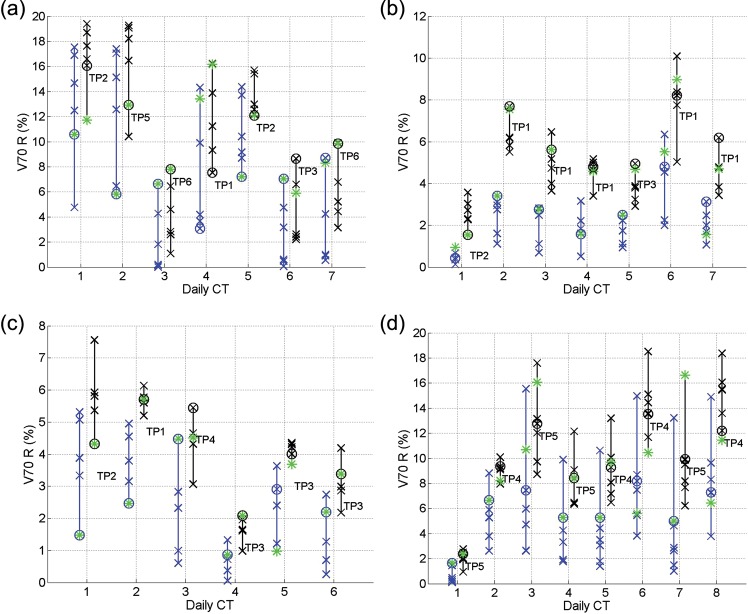
V70 values for the rectum calculated from applying all the SP plans (blue crosses) and GP–OAR plans (black crosses) in the libraries of (**a**) patient 1, (**b**) patient 2, (**c**) patient 3, and (**d**) patient 4. Daily chosen PoDs are marked with circles, and the best plans in the libraries are marked with asterisks.

From Figs 4–7, the V50 and V70 values of the OARs calculated from applying all the SP plans (blue crosses) and GP–OAR plans (black crosses) are illustrated. Daily plans PoDs_SP_ and PoDs_GP–OAR_ are marked with circles in blue and black, respectively, and the best plans in the libraries are marked with asterisks. In all daily cases but one, the dose delivered to the bladders by the chosen PoD_GP–OAR_s is lower or similar (V50 differences and V70 differences within 5%) compared with the dose delivered by the best plans from the GP–OAR libraries. For the exceptional daily case 4 of patient 1, V50 and V70 values of the bladder calculated from applying the PoD_GP–OAR_ are 6.8% and 5.4% higher than those calculated from applying the best plan, respectively. The dose received by the bladders from applying all the GP–OAR plans in the libraries varies from patient to patient, with the mean V50 ranges of 19.7%, 14.1%, 6.2% and 3.6%, and the mean V70 ranges of 15.2%, 12.8%, 4.7% and 2.5% for patients 1, 2, 3 and 4, respectively. The maximum V50 values of the bladders are 74.3%, 43.1%, 23.4% and 15.9% for the four patients, respectively, and the maximum V70 values are 53.5%, 36.6%, 15.2% and 10.6%, respectively. When chosen by similarity measures, in all but one case the PoDs_GP–OAR_ provide V50 values of the bladders < 50%, as restricted during treatment planning. In daily case 7 of patient 1, the V50 of the bladder calculated from applying the PoD_GP–OAR_ is around 59%. When considering dose received by the rectum, all PoDs_GP–OAR_ deliver less or similar dose compared with the best plans in the library. The mean ranges of V50 values of the rectum calculated from applying the whole GP–OAR plan libraries are 7.7%, 3.5%, 2.1% and 7.0% for patients 1, 2, 3 and 4, respectively, and the mean ranges of V70 values are 13.8%, 6.3%, 5% and 13.7%, respectively. The maximum V50 values of the rectum are 24.2%, 13.9%, 10.4% and 24.0% for the four patients, respectively, which indicates that the dose received by the rectum when applying the GP–OAR plans always stays within the constraints.

When comparing the chosen PoDs_GP–OAR_ and the PoDs_SP_ in each simulated daily case, V50 values of the bladder calculated from applying the former plans are similar to those calculated from applying the latter ones, with a maximum V50 increase of 2.2% in daily case 4 of patient 3 and a maximum V70 increase of 2.2% in daily case 7 of patient 1 and daily case 4 of patient 3. In 19 out of 28 cases the PoDs_GP–OAR_ deliver a similar dose to the rectum compared with the PoDs_SP_. In daily cases 1, 2, 4 and 5 of patient 1, daily case 2 of patient 2 and daily cases 3, 6, 7 and 8 of patient 4, the V50 and/or V70 differences of the rectum calculated when applying the PoDs_GP–OAR_ and the PoDs_SP_ are > 5%, with maximum V50 differences of 8.1%, 5.4% and 6.1% for patients 1, 2 and 4, respectively, and with maximum V70 differences of 7.1% and 5.3% for patients 1 and 4, respectively.

## DISCUSSION

Similarity comparison between planning images and daily scans is a good way to choose a suitable if not the best plan from a treatment plan library: most daily chosen PoDs_SP_ and PoDs_GP–OAR_ give the best target coverage, or coverage similar (V95 differences < 5%) to that provided by the best plans in the libraries. Besides, dose delivered to the OARs by the best plans and the PoDs are mostly similar (V50 differences < 5%). Despite the wide V95 range of a target when applying a whole library, V95 values of the prostates calculated from applying the PoDs_SP_ are mostly > 90%, and in 9 out of 28 cases > 95%. When calculated from applying the PoDs_GP–OAR_, target coverage is sufficient in all but three cases, with V95 values of the targets > 95%. In the exceptional daily cases 2, 3 and 5 of patient 2, the V95 values of the targets calculated from applying the PoDs_GP–OAR_ are 91.1%, 93.1% and 94.5%, respectively. For the first two daily cases, even the best plans in the GP–OAR libraries are not able to provide sufficient target coverage. As a matter of fact, the target volumes on these two daily images are the largest ones for that patient, and are about 1.3 times the size of the mean prostate volume. Thus, a union of the prostate contours on the other images might be not able to provide enough coverage for the large prostate in these two daily cases.

When applying all the SP plans in the libraries, the V95 ranges of the targets are larger for patients 2 and 4 compared with patients 1 and 3. This might be due to larger target volume variations for the former two patients according to Table 1. On the other hand, the V95 ranges of the targets calculated from applying the GP–OAR plans for the four patients become smaller and closer to each other compared with the ranges when applying the SP plans. In addition, the improvement in target coverage of the GP–OAR plans compared with the SP plans is obvious, and the maximum V95 values of the targets calculated from applying the best GP–OAR plans are significantly higher than those calculated from applying the best SP plans. This indicates that the GP–OAR plans are able to cover interfractional variations to a larger extent compared with plans generated with a single planning image only.

Considering dose delivered to the OARs, the V50 ranges of bladder and rectum calculated from applying the SP plans and GP–OAR plans are similar. Besides, one cannot find significant differences in OAR sparing between PoD_GP–OAR_s and PoDs_SP_. The V50 differences calculated for the bladder between applying PoD_GP–OAR_s and PoDs_SP_ are mostly within 5%. For the rectum, there are nine cases in which the differences are > 5%. Nevertheless, the V50 values for the rectum calculated from applying the GP–OAR plans are always < 25% and are less than the constraints of 50%. Thus, we conclude that plans generated on the PTV GP–OARs provide slightly increased but comparable OAR sparing to the ones optimized on the SP volumes. We note that there are several cases in which the PoD_GP–OAR_s deliver less dose to the bladders compared with the PoDs_SP_. This could be explained by fact that the exclusion of the OARs from the union of the prostate volumes includes the overlapping part with the prostate.

As shown in Table 1, the volume variation percentages of the prostates are 23% and 22% for patients 2 and 4, respectively. On the other hand, the variation percentages are merely 6% for the other two patients. Accordingly, in Fig. 3 one can see that the V95 ranges of the targets of patients 2 and 4 are generally larger than those of patients 1 and 3. The bladder volume variation for patients 1 and 2 and the rectal gas volume variation for patients 1, 2 and 4 are higher than those of the other patients. Meanwhile, the V50/V70 ranges are larger for these patients, as shown in Figs 4–7. Protocols regulating the sizes of OARs can help reduce the organ variations, and hence improve the performance of PoD_GP–OAR_s. To extend the proposed method further, a global OAR volume can be determined based on multiple images to guide the PTV definition and treatment planning so that the OARs can be better spared.

The minimum number of CT datasets necessary for generation of a GP–OAR plan library needs to be further considered. In this study, patient 3 has the smallest size of plan library of the four patients. With six images available and each one picked as a daily scan in turn, GP–OAR volumes are generated from the prostate contours delineated on the five remaining CT datasets. For this patient, the PoD_GP–OAR_s are able to provide satisfactory target coverage, and five CT datasets are enough to represent the possible variations. On the other hand, as listed in Table 1, the smallest volume variations of the prostate, bladder and rectum gas of the four patients can be found in patient 3. More datasets for patients with larger variations might be needed to generate the patient-specific treatment volume. For example, patient 2 has the largest prostate and OAR volume variations. Although generated from six CT datasets, none of the GP–OARs can provide sufficient target coverage in daily cases 2 and 3. The number of CT datasets needed for GP–OAR generation varies from patient to patient, largely depending on the level of interfractional variations of the targets and OARs. The commonly used modalities in image guidance for prostate cases are ultrasonography, portal imaging, kV/MV CT or cone-beam CT [16]. To generate a treatment-planning library beforehand, multiple CT scans must be acquired and contoured, and treatment planning on each image should be carried out. Besides, daily imaging is also necessary for the PoD selection. This means a higher imaging dose for the patients, increased workload for the physicians, and lower patient throughput for the hospital. Although the advantage of the proposed method for the target coverage and OAR sparing is demonstrated in this study, the implementation will be debatable as it might be too costly and time-consuming. One suggestion is that this method can be used in hypo-fractionated treatments where higher accuracy is required.

In the previous PoD studies, to cover possible anatomy variations inside a patient, a plan library can be generated either on one planning image with different PTV–CTV margins, or from multiple images taken on different days. These studies have shown promising results, allowing greater sparing of healthy tissues compared with using repositioning only while keeping sufficient coverage of a target. However, a limiting factor for practical implementation is that the selection of a daily plan depends on physician observation and intervention. As the adaptation is performed online, the plan selection procedure cannot be an extended process. In a short time window for observation or contouring, the judgement of a physician might be compromised. To facilitate the selection of a daily plan, we deploy the properties of images themselves. An assumption is made that the difference between two images is correlated with patient's anatomy differences between the two days on which the images are taken. Thus, a suitable plan can be chosen from a plan library by similarity comparison between a daily image and the planning images.

In our study, the treatment plans are not optimized according to the clinical standard. To exaggerate the dose impact of applying different plans, a small PTV–CTV margin is applied to the target. As a proof-of-principle study, only physical dose is considered in treatment planning. With a wide range of dose coverage for the target of the whole treatment plan library, the advantage of choosing the PoD has been demonstrated. The authors expect similar results when the biological effectiveness is taken into account. In addition, this feasibility study is performed with single fraction simulation. In radiotherapy, the prescribed dose is delivered via multiple fractions, reducing the dose inhomogeneity and keeping the total dose to the target. Nevertheless, the daily chosen PoD generally provides similar if not the best dose coverage to the target. By applying the PoD instead of the same plan every fraction, one could anticipate a higher cumulative dose to the target over the treatment course.

## CONCLUSION

This study uses a patient-specific PTV to deal with interfractional target motion. Instead of applying a generic margin outside the CTV, possible variations of a target are accounted for by a union of target volumes on multiple planning images. To minimize the dose impact of the enlarged planning volume on the surrounding critical organs, voxels which belong to the OARs are excluded from the union. The feasibility and effectiveness of the proposed PTV is tested in a retrospective treatment-planning study, and the simulated daily cases are adapted with the PoD method. The results show that the chosen daily plans optimized on the proposed PTV are able to provide satisfactory target coverage, while the dose delivered to the OARs are slightly increased but comparable to that when applying daily plans optimized from a single planning image.

## FUNDING

This work was supported by the PARTNER (Particle Training Network for European Radiotherapy) project of the European Community's Seventh Framework Programme (FP7/2007–2013) under Grant Agreement No. 215840-2.
